# Supplementary material for: SARS-CoV-2 shedding dynamics across the respiratory tract, sex, and disease severity for adult and pediatric COVID-19
Source: eLife. 2021 Aug 20;10:e70458. doi: 10.7554/eLife.70458 (PMC8504968; doi:10.7554/eLife.70458)
Supplement: Figure 1—source data 2. [file elife-70458-fig1-data2.docx]

Figure 1— Source Data 2. Search strategy used for EMBASE.

| Database: **Embase** 1974 to 20 Nov 2020 | |
| --- | --- |
| **#** | **Searches** |
| 1 | coronavirus infection/ or severe acute respiratory syndrome/ |
| 2 | COVID 19/ |
| 3 | coronavirus disease 2019/ |
| 4 | severe acute respiratory syndrome coronavirus 2/ or SARS coronavirus 2/ |
| 5 | SARS coronavirus/ or betacoronavirus/ |
| 6 | “influenza a virus (h1n1)”/ |
| 7 | 2009 h1n1 influenza/ or “influenza a (h1n1)”/ |
| 8 | (coronavirus* or corona virus* or betacoronavirus* or OC43 or NL63 or 229E or HKU1 or HcoV* or ncov* or covid* or sars-cov* or sarscov* or Sars-coronavirus* or Severe Acute Respiratory Syndrome* or Sudden acute respiratory syndrome*).tw,kw. |
| 9 | (2019nCov* or 2019-novel CoV or corona or covid19 or ((novel or new or nouveau) adj2 (CoV or Pandemi*))).tw,kw. |
| 10 | (pneumonia.tw,kw. Or exp pneumonia/) and (Wuhan or Hubei).tw,kw. |
| 11 | (covid or SARS or H1N1 or coronavirus).ox. |
| 12 | (“A/H1N1*” or H1N1* or pdm09 or ((influenza or virus or pandemic) adj4 “2009”) or influenza A or swine flu).tw,kw. |
| 13 | 1 or 2 or 3 or 4 or 5 or 6 or 7 or 8 or 9 or 10 or 11 or 12 |
| 14 | nose smear/ |
| 15 | smear/ or nose smear/ or sputum smear/ |
| 16 | *sputum/ |
| 17 | *nasopharynx/ or *pharynx/ |
| 18 | throat culture/ |
| 19 | saliva analysis/ |
| 20 | *oropharynx/ |
| 21 | exp nasopharyngeal aspiration/ |
| 22 | real time polymerase chain reaction/ or real time reverse transcription polymerase chain reaction/ or reverse transcription polymerase chain reaction/ |
| 23 | (clinical adj2 (sample* or specimen*)).tw,kw. |
| 24 | ((respiratory adj3 (specimen* or sample* or swab*)) or sputum or nares or endotrachea* or endotrache* or endotra* or ((nasal or oral* or throat) adj3 (swab* or sample* or smear* or specimen*)) or NPS or OPS or ((endotrachea* or endotracheal*) adj2 aspirat*) or NPA or ETA or (deep adj4 saliva) or POS or “swab sample*” or “flocked swab*”).tw,kw. |
| 25 | (“RT-PCR” or “RTPCR” or “ddPCR” or “polymerase chain reaction”).tw,kw. |
| 26 | *2009 H1N1 influenza/di or coronavirus disease 2019/di or Coronavirus infection/di |
| 27 | 14 or 15 or 16 or 17 or 18 or 19 or 20 or 21 or 22 or 23 or 24 or 25 or 26 |
| 28 | 13 and 27 |
| 29 | virus load/ |
| 30 | virus shedding/ |
| 31 | (vir* load* or vir* shed* or vir* burden or vir* titer* or vir* titre* or (vir* adj2 count*)).tw,kw. |
| 32 | (((copies or copy) adj2 (ml* or milli* or microl*)) or ((RNA* or vir*) adj2 concentration*)).tw,kw. |
| 33 | ((calibration adj1 curve*) or (standard adj1 curve*)).tw,kw. |
| 34 | ((ct* adj1 value*) or cycle threshold or (copies adj2 test*) or (copy adj2 test*) or ((copy or copies) adj2 number*)).tw,kw. |
| 35 | (“copy/m*” or “copies/m*” or “copy/test*” or “copies/test*”).tw,kw. |
| 36 | ((test or diagnos*) adj2 sensitiv*).tw,kw. |
| 37 | 29 or 30 or 31 or 32 or 33 or 34 or 35 or 36 |
| 38 | 28 and 37 |
| 39 | exp animal/ not human/ |
| 40 | 38 not 39 |
