# Supplementary material for: SARS-CoV-2 shedding dynamics across the respiratory tract, sex, and disease severity for adult and pediatric COVID-19
Source: eLife. 2021 Aug 20;10:e70458. doi: 10.7554/eLife.70458 (PMC8504968; doi:10.7554/eLife.70458)
Supplement: Figure 1—source data 4. [file elife-70458-fig1-data4.docx]

Figure 1— Source Data 4. Search strategy used for Web of Science Core Collection.

| **Web of Science Core Collection**  Database: **Web of Science Core Collection** up to 20 Nov 2020 | |
| --- | --- |
| **#** | **Searches** |
| #1 | TOPIC: ((coronavirus* or “corona virus*” or betacoronavirus* or OC43 or NL63 or 229E or HKU1 or HcoV* or ncov* or covid* or “sars-cov*” or sarscov* or “Sars-coronavirus*” or “Severe Acute Respiratory Syndrome*” or “sudden acute respiratory syndrome*” or “2019-ncov*” or 2019nCov* or “2019-novel CoV” or corona or ((novel or new or nouveau) NEAR/2 (CoV or Pandemi*) ) OR (pneumonia and (Wuhan or Hubei) ) or “A/H1N1*” or H1N1* or pdm09 or ((influenza or virus or pandemic) NEAR/4 “2009”) or “influenza A” or “swine flu”))  Indexes=SCI-EXPANDED, SSCI, A&HCI, CPCI-S, CPCI-SSH, ESCI Timespan=All years |
| #2 | TOPIC: (((respiratory NEAR/3 (specimen* or sample* or swab*) ) or sputum or nares or endotrachea* or endotrache* or endotra* or ((nasal or oral* or throat) NEAR/3 (swab* or sample* or smear* or specimen*) ) or NPS or OPS or ((endotrachea* or endotracheal*) NEAR/2 aspirat*) or NPA or ETA or (deep NEAR/4 saliva) or POS or “swab sample*” or “flocked swab*” or (clinical NEAR/2 (sample* or specimen*) ) or “RT-PCR” or “RTPCR” or “ddPCR” or “polymerase chain reaction”))  Indexes=SCI-EXPANDED, SSCI, A&HCI, CPCI-S, CPCI-SSH, ESCI Timespan=All years |
| #3 | TOPIC: ((“vir* load*” or “vir* shed*” or “vir* burden” or “vir* titer*” or “vir* titre*” or (vir* NEAR/2 count*) or ((copies or copy) NEAR/2 (ml or mls or milli* or microl*) ) or ((RNA* or vir*) NEAR/2 concentration*) or (calibration NEAR/1 curve*) or (standard NEAR/1 curve*) or “ct value*” or “cycle threshold” or ((copies or copy) NEAR/2 test*) or ((copy or copies) NEAR/2 number*) or “copy/m*” or “copies/m*” or “copy/test*” or “copies/test*” or ((test or diagnos*) NEAR/2 sensitiv*)))  Indexes=SCI-EXPANDED, SSCI, A&HCI, CPCI-S, CPCI-SSH, ESCI Timespan=All years |
| #4 | #3 AND #2 AND #1  Refined by: [excluding] WEB OF SCIENCE CATEGORIES: ( VETERINARY SCIENCES )  Indexes=SCI-EXPANDED, SSCI, A&HCI, CPCI-S, CPCI-SSH, ESCI Timespan=All years |
